# Supplementary material for: In vitro one-pot construction of influenza viral genomes for virus particle synthesis based on reverse genetics system
Source: PLoS One. 2024 Nov 8;19(11):e0312776. doi: 10.1371/journal.pone.0312776 (PMC11548778; doi:10.1371/journal.pone.0312776)
Supplement: S1 Fig — Each plasmid was constructed by assembling a vector fragment containing the oriC sequence, the human RNA polymerase I promoter (green, Pol I promoter), and the mouse RNA polymerase I terminator sequence (orange, Pol I terminator), together with a cDNA fragment (red) encoding each influenza viral segment in negative-sense orientation. PCR amplification was performed across the junction between the vector and the viral cDNA fragment using the primer pairs designed to bind to each fragment. “Fw” indicates the forward primer, and “Rv” indicates the reverse primer. (PDF) [file pone.0312776.s001.pdf]

A

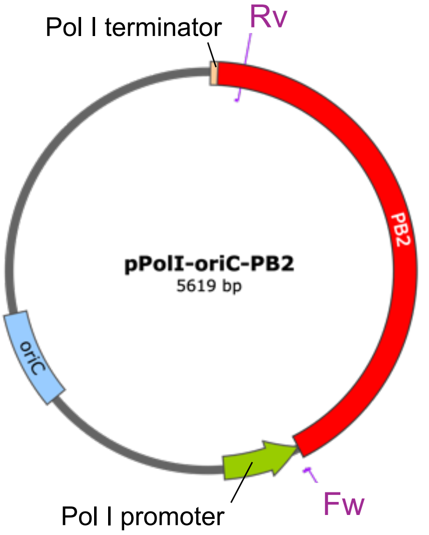

Primer name	Nucleotide sequence (5' -> 3')
Fw	AATAACCCGGCGGCCCAAAATG
Rv	TGGCCATATGGTCCACGGTG

B

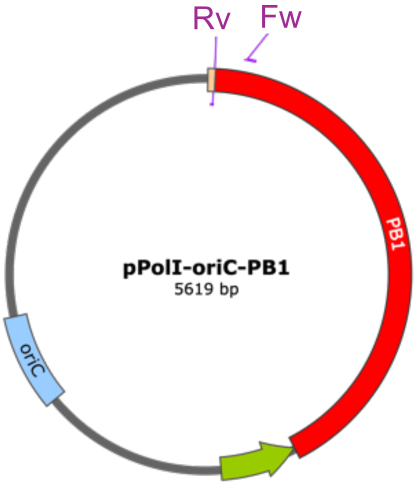

Primer name	Nucleotide sequence (5' -> 3')
Fw	ACAGGATACACCATGGATACTGTCAACAGG
Rv	CCCCCCCAACTTCGGAGGTC

C

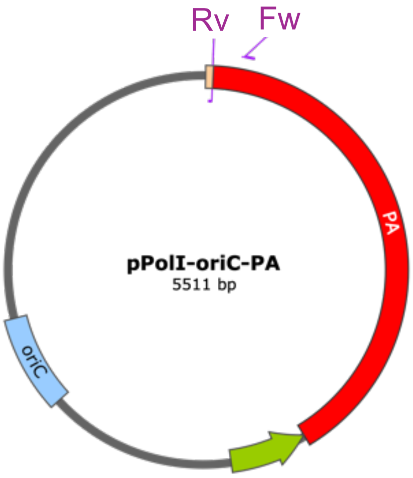

Primer namec	Nucleotide sequence (5' -> 3')
Fw	GCAGCAATATGCACTCACTTGGAAGTATGC
Rv	CCCCCCCAACTTCGGAGGTC

D

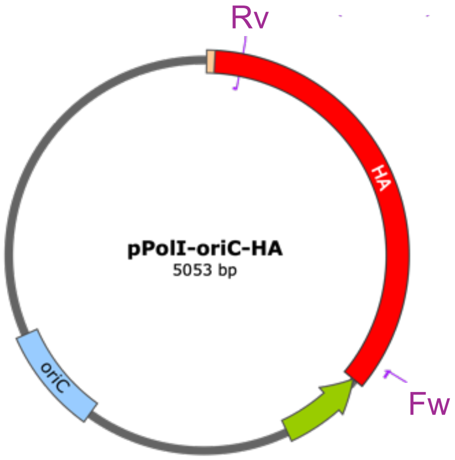

Primer namec	Nucleotide sequence (5' -> 3')
Fw	GGCAATCAGTTTCTGGATGTG
Rv	GTTCGCATGGTAGCCTATAC

E

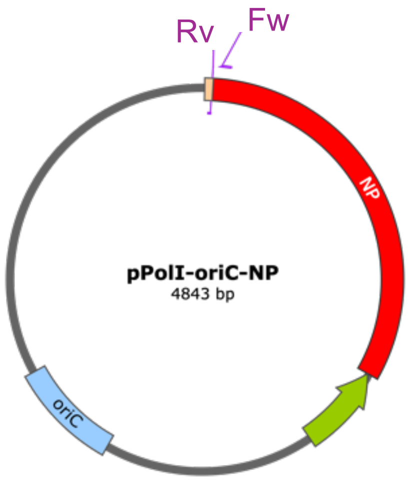

Primer name	Nucleotide sequence (5' -> 3')
Fw	GAGTGACATCAAAATCATGGCGTCTCAAGG
Rv	CCCCCCCAACTTCGGAGGTC

F

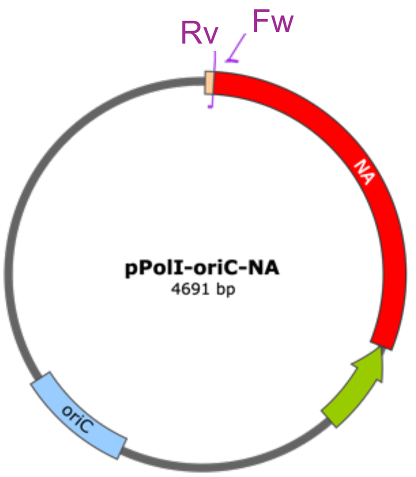

Primer namec	Nucleotide sequence (5' -> 3')
Fw	CCATTGGATCAATCTGTCTGGTAGTCGGAC
Rv	CCCCCCCAACTTCGGAGGTC

G

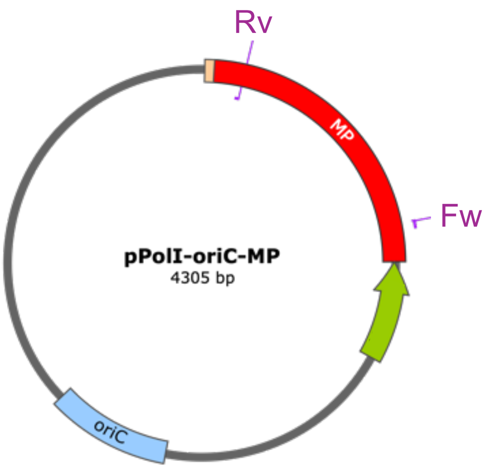

Primer name	Nucleotide sequence (5' -> 3')
Fw	GGCCTTCTACGGAAGGAGTG
Rv	CAAGTCTCTGTGCGATCTCG

H

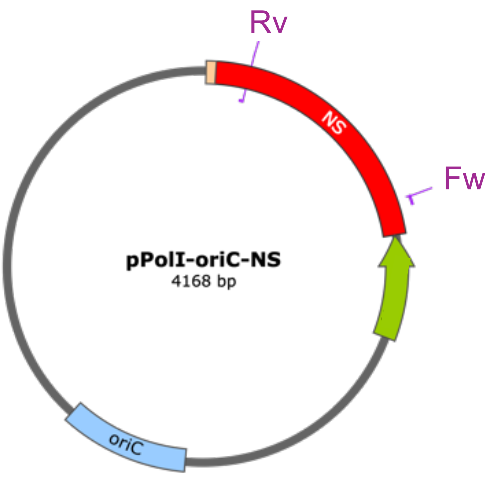

Primer namec	Nucleotide sequence (5' -> 3')
Fw	GAGCAAATAACATTTATGCAAGCC
Rv	GGGCATCGCCTAGTTCTTGG

S1 Fig. The detail information of primers used for verifying the construction of eight pPolI-oriC plasmids. Each plasmid was constructed by assembling a vector fragment containing the oriC sequence, the human RNA polymerase I promoter (green, Pol I promoter), and the mouse RNA polymerase I terminator sequence (orange, Pol I terminator), together with a cDNA fragment (red) encoding each influenza viral segment in negative-sense orientation. PCR amplification was performed across the junction between the vector and the viral cDNA fragment using the primer pairs designed to bind to each fragment. “Fw” indicates the forward primer, and “Rv” indicates the reverse primer.
