# Supplementary material for: In vitro one-pot construction of influenza viral genomes for virus particle synthesis based on reverse genetics system
Source: PLoS One. 2024 Nov 8;19(11):e0312776. doi: 10.1371/journal.pone.0312776 (PMC11548778; doi:10.1371/journal.pone.0312776)
Supplement: S2 Fig — Each plasmid was constructed by assembling a vector fragment containing the oriC sequence, an RNA polymerase II promoter (pink, Pol II promoter, e.g., chicken β-actin promoter), a polyadenylation sequence (orange, PolyA, e.g., the rabbit β-globin polyadenylation sequence), and a gene sequence (black, chimeric intron) that includes introns from the chicken β-actin gene and rabbit β-globin gene, together with a cDNA fragment (red) encoding each viral protein. PCR amplification was performed across the junction between the vector and the viral cDNA fragment using the primer pairs designed to bind to each fragment. “Fw” indicates the forward primer, and “Rv” indicates the reverse primer. (PDF) [file pone.0312776.s002.pdf]

A

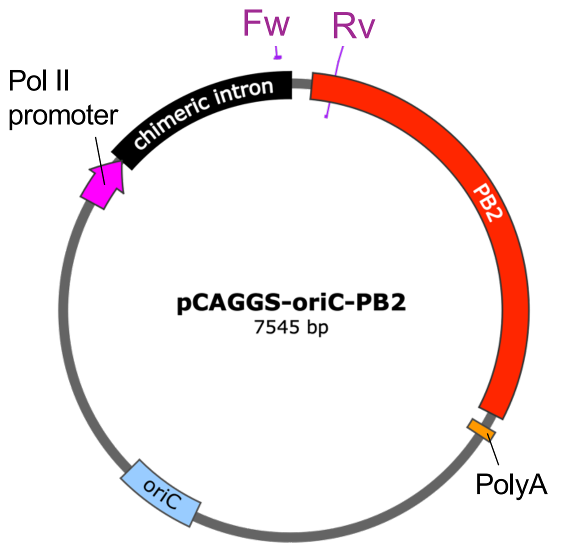

Primer name	Nucleotide sequence (5' -> 3')
Fw	GGCTTCTGGCGTGTGACC
Rv	TGGCCATATGGTCCACGGTG

B

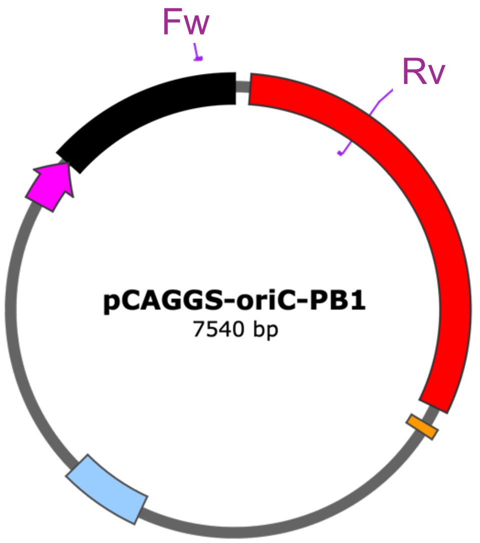

Primer name	Nucleotide sequence (5' -> 3')
Fw	GAGGGCCTTCGTGCGTC
Rv	TTACCCATTGTTCTCTGTGTTATCATTTTCT TAG

C

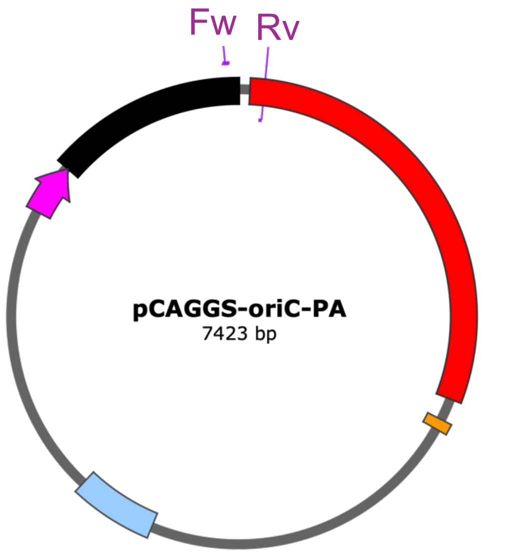

Primer name	Nucleotide sequence (5' -> 3')
Fw	GGCTTCTGGCGTGTGACC
Rv	TTTCCGCAAGCTCGACAATC

D

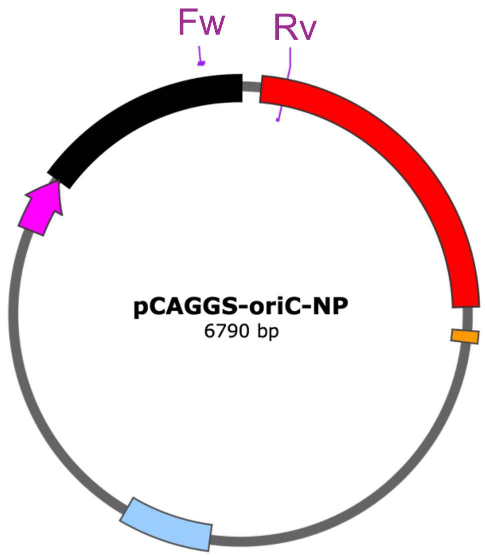

Primer name	Nucleotide sequence (5' -> 3')
Fw	GAGGGCCTTCGTGCGTC
Rv	TCAGTGGCATTCTGGCGTTC

S2 Fig. The detail information of primers used for verifying the construction of pCAGGS-oriC plasmids. Each plasmid was constructed by assembling a vector fragment containing the oriC sequence, an RNA polymerase II promoter (pink, Pol II promoter, e.g., chicken β-actin promoter), a polyadenylation sequence (orange, PolyA, e.g., the rabbit β-globin polyadenylation sequence), and a gene sequence (black, chimeric intron) that includes introns from the chicken β-actin gene and rabbit β-globin gene, together with a cDNA fragment (red) encoding each viral protein. PCR amplification was performed across the junction between the vector and the viral cDNA fragment using the primer pairs designed to bind to each fragment. “Fw” indicates the forward primer, and “Rv” indicates the reverse primer.
