# Supplementary figures and images for: In vitro one-pot construction of influenza viral genomes for virus particle synthesis based on reverse genetics system
Source: PLoS One. 2024 Nov 8;19(11):e0312776. doi: 10.1371/journal.pone.0312776 (PMC11548778; doi:10.1371/journal.pone.0312776)

Fig 2B (left)

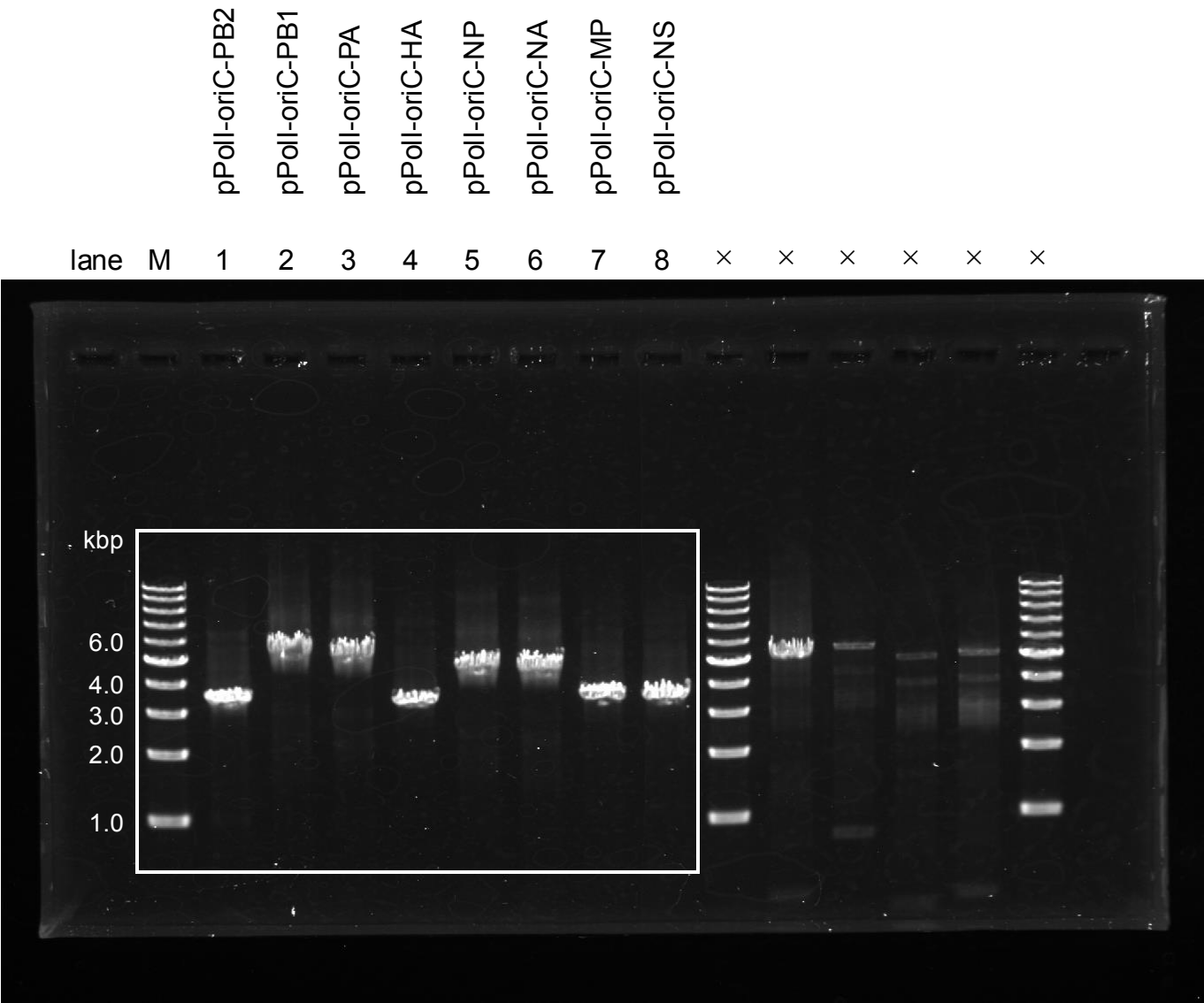

Fig 2B (right)

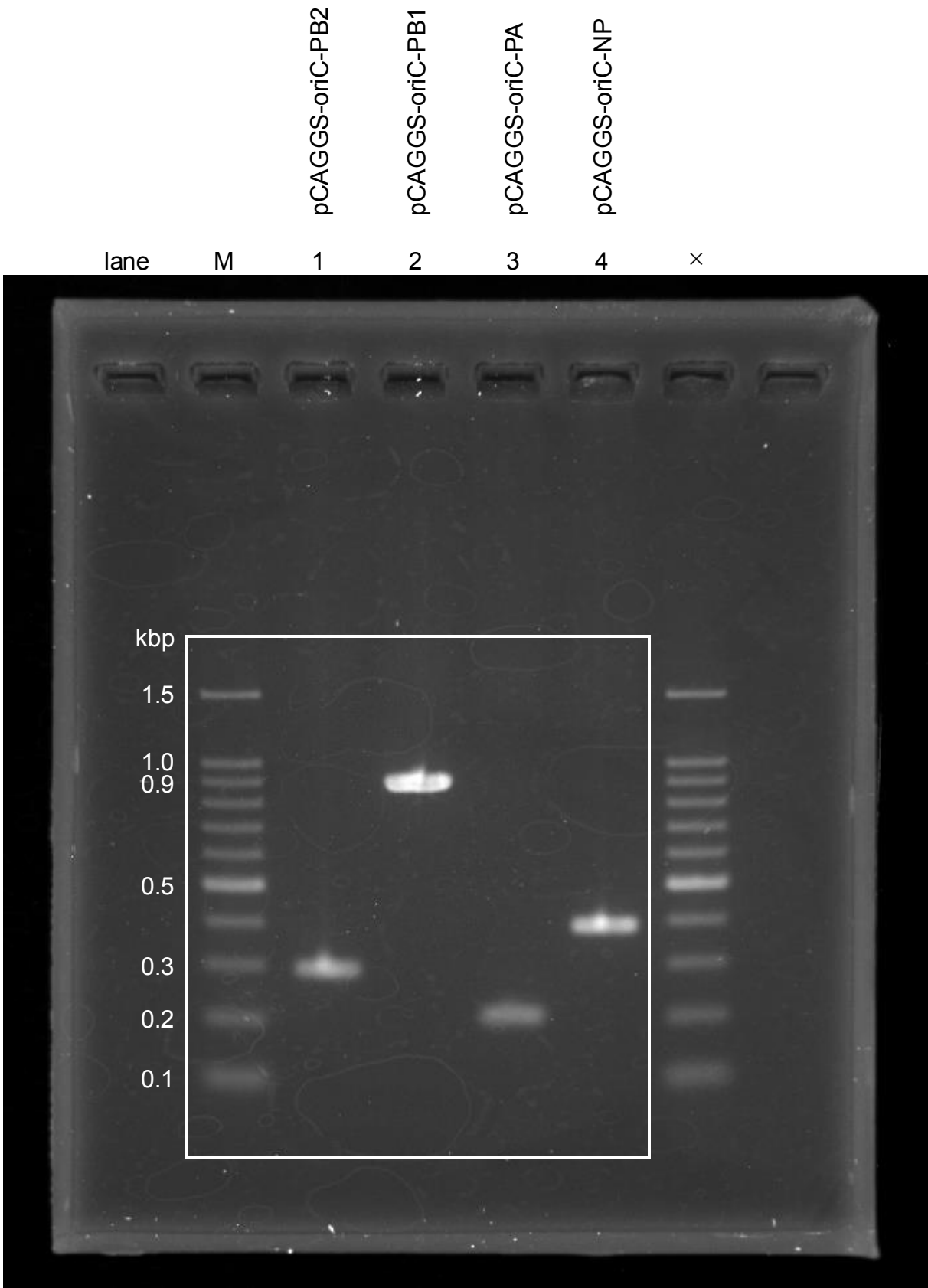

Fig 6B

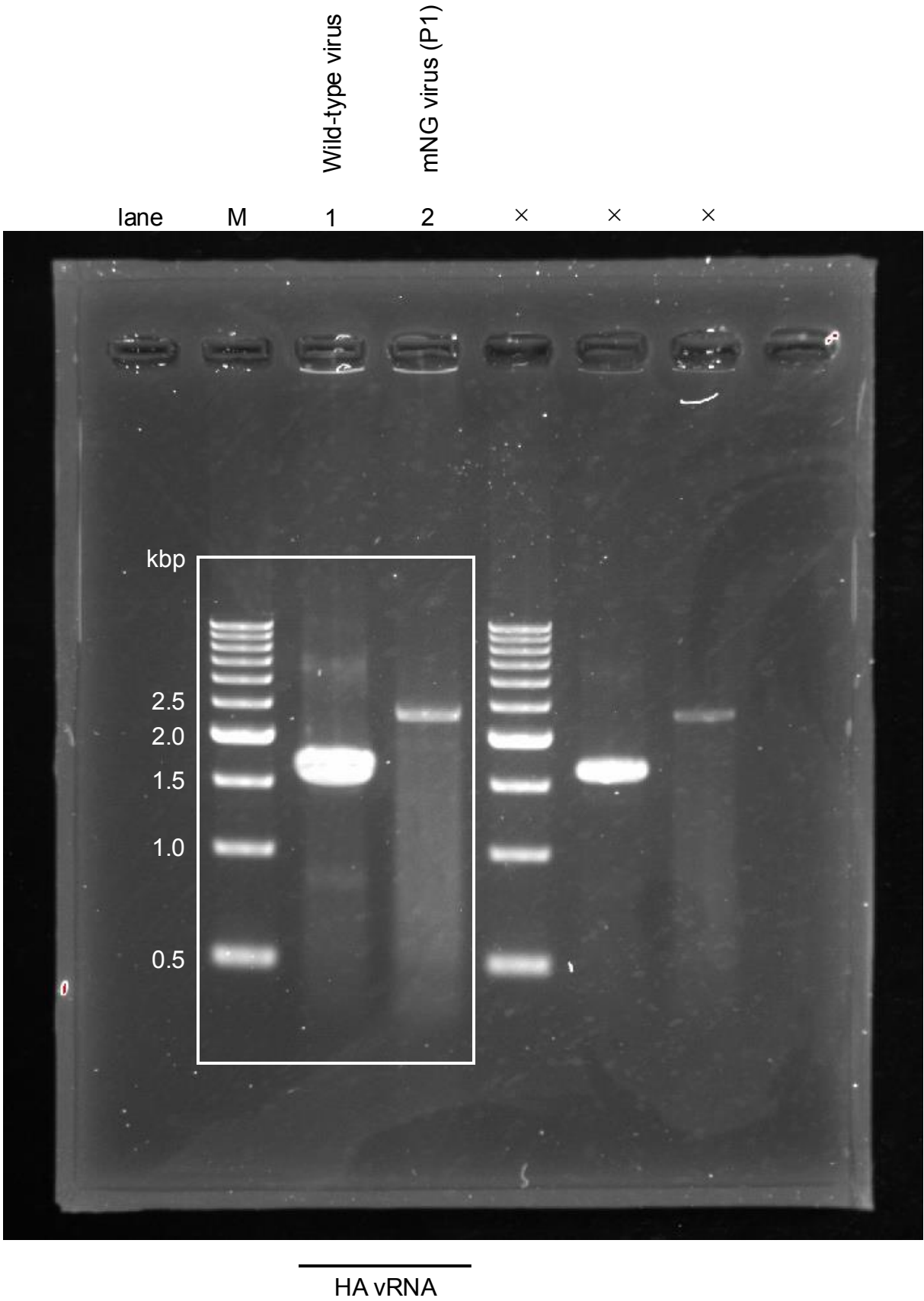

Supplement: S1 Raw image — Agarose gel electrophoreses for verification of plasmid construction using the IVOC method (Raw Fig 2B) and demonstration of recombinant virus particle synthesis using the IVOC approach (Raw Fig 6B). (PDF) [file pone.0312776.s004.pdf]
