# Supplementary material for: In vitro one-pot construction of influenza viral genomes for virus particle synthesis based on reverse genetics system
Source: PLoS One. 2024 Nov 8;19(11):e0312776. doi: 10.1371/journal.pone.0312776 (PMC11548778; doi:10.1371/journal.pone.0312776)
Supplement: Attachment — Submitted filename: Response to Reviewers.pdf [file pone.0312776.s005.pdf]

Response to Reviewers (PONE-D-24-30909)

In vitro one-pot construction of influenza viral genomes for virus particle synthesis based on reverse genetics system

Ryota Tanaka[1], Kenji Tamao[1], Mana Ono[1], Seiya Yamayoshi[2,3], Yoshihiro Kawaoka[2,3,4,5], Masayuki Su'etsugu[6], Hiroyuki Noji[1], Kazuhito V. Tabata[1*]

We sincerely appreciate the reviewers for the careful reading and constructive comments. According to the comments, we have revised the manuscript.

Reviewers' comments are shown in *blue Italic*.

Journal Requirements

1. Please ensure that your manuscript meets PLOS ONE's style requirements, including those for file naming.

We confirmed and revised ensure that it conformed to the PLOS ONE style.

2. Thank you for stating the following financial disclosure:
"the Japan Science and Technology Agency for Core Research for Evolutional Science and Technology, JST CREST, Japan (JPMJCR18S6 and JPMJCR22N2), The Ogasawara Toshimasa Memorial Foundation"
Please state what role the funders took in the study. If the funders had no role, please state: "The funders had no role in study design, data collection and analysis, decision to publish, or preparation of the manuscript."
If this statement is not correct you must amend it as needed.
Please include this amended Role of Funder statement in your cover letter; we will change the online submission form on your behalf.

3. Please note that funding information should not appear in the Acknowledgments section or other areas of your manuscript. We will only publish funding information present in the Funding Statement section of the online submission form. Please remove any funding-related text from the manuscript.

Regarding requirements 2 and 3, we have removed the information on the research

funding agencies from the acknowledgements and transferred it to the online submission form. We have also added a description of the role of the research funding agencies.

4. PLOS ONE now requires that authors provide the original uncropped and unadjusted images underlying all blot or gel results reported in a submission's figures or Supporting Information files. This policy and the journal's other requirements for blot/gel reporting and figure preparation are described in detail at https://journals.plos.org/plosone/s/figures#loc-blot-and-gel-reporting-requirements and https://journals.plos.org/plosone/s/figures#loc-preparing-figures-from-image-files. When you submit your revised manuscript, please ensure that your figures adhere fully to these guidelines and provide the original underlying images for all blot or gel data reported in your submission. See the following link for instructions on providing the original image data: https://journals.plos.org/plosone/s/figures#loc-original-images-for-blots-and-gels.

In your cover letter, please note whether your blot/gel image data are in Supporting Information or posted at a public data repository, provide the repository URL if relevant, and provide specific details as to which raw blot/gel images, if any, are not available. Email us at plosone@plos.org if you have any questions.

We have uploaded all the original images of the electrophoresis results used in this paper to Raw_gel_images.pdf. We have also prepared the sequence data for Fig. S3 and uploaded these together to the repository (https://osf.io/9fyeu/). This information is also included in the main text.

5. Please review your reference list to ensure that it is complete and correct. If you have cited papers that have been retracted, please include the rationale for doing so in the manuscript text, or remove these references and replace them with relevant current references. Any changes to the reference list should be mentioned in the rebuttal letter that accompanies your revised manuscript. If you need to cite a retracted article, indicate the article's retracted status in the References list and also include a citation and full reference for the retraction notice.

We checked the reference list again to make sure everything was correct.

We have made some changes to the text to make it easier to read, such as changing IVOC to IVOC approach. These changes are highlighted in the text, so please review them. If you feel the changes are not appropriate, we will make the necessary changes again.

Reviewer #1

Specific comments:

1. In the abstract: It is unclear to the reviewer why the authors expect that this method "potentially advance further understanding of influenza viruses…".

At the end of the abstract, the following text was included to explain how the IVOC method can be used in influenza virus research.

This method enables the pathogenicity analysis and vaccine development using genetically modified viruses, and it is expected to allow for faster analysis of newly emerging variants than ever before. Furthermore, its application to other RNA viruses is also expected.

2. In the introduction: Please clarify that only influenza A viruses have caused known pandemics.

We have made the following changes to the introduction section you pointed out.

In particularthe 20th century, influenza A viruses caused there were 3 three pandemics in the 20th century (9), and in the 21st century, there was a pandemic was caused by a swine-origin influenza A virus in 2009(10). Considering the potential emergence of new pathogenic viruses(11), it is urgent to advance our biological understanding of the influenza A virus through research and the development of tools to counter its threat to humanity.

General comments:

1. The reviewer understood the IVOC approach to mean that the authors generated pPolI and pCAGGS plasmids with an E. coli oriC. For clarity, the authors should consider naming these modified plasmids, e.g. as pPolI-oriC, rather than referring to them as "these plasmids". In addition, Figure 1 should be adjusted accordingly to distinguish the plasmids with oriC (upper part of the figure) from the conventional pPolI and pCAGG plasmids without the oriC.

As you suggested, in order to make the names of the plasmids easier to understand, we have changed the plasmids used as templates for PCR to pPolI and pCAGGS, and the plasmids that have oriC to pPolI-oriC and pCAGGS-oriC. Therefore, we have made several corrections to the main text. In addition, we have made corrections to Figures 1 and 5 to distinguish between plasmids that do and do not have oriC. Please confirm the relevant sections.

2. In the Materials and Methods section: Authors should provide the manufacturers of all reagents, especially the E. coli enzyme mix required for the IVOC method. Is it commercially available?

All the reagents required for RA-RCR were prepared by Su'etsugu et al. The text has been modified as follows to make this clear.

Plasmid construction using the RA-RCR system followed the protocol outlined in a previous report(30). The RA mix and RCR mix required for the RA-RCR system were prepared by Su'etsugu et.al according to that report(30).

3. Because it is unclear to the reviewer why segments of similar length (e.g. PB2 and PB1) would generate a different amplicon size, the authors should provide a list of the primer pairs (and show were they bind in the plasmid backbone) used to generate the PCR products shown in Figure 2B.

The primer sets used in the PCR in Fig. 2B are listed in the Supporting Information as Fig. S1 and S2, along with the sequences and annealing positions for each plasmid. This is also shown in the legend for Fig. 2. As a result of this change, the previous Fig. S1 has been moved to Fig. S3.

4. In this study, the authors generated PR8 viruses using available plasmids as a template. The authors should comment on whether or not they tested isolated vRNA from infectious cell culture supernatant as a template for the IVOC approach. The authors should also mention whether or not this approach can be used to rapidly generate a plasmid rescue system for virus isolates from infectious patient/animal material.

In this study, we did not perform IVOC using vRNA isolated from clinical specimens. However, because general reverse genetics methods reconstitute viruses from vRNA derived from clinical specimens, we believe that IVOC is also possible. We will perform such experiments in the future. In this regard, we have also added the following text to the main text.

In this study, viral RNA isolated and purified from infectious cell culture supernatants or patient/animal samples was not tested as a template in the IVOC approach. However, it is anticipated that this would be feasible within the IVOC system, as viral RNA has successfully served as a template for virus production in the conventional methods.
